# Cabazitaxel multiple rechallenges in metastatic castration‐resistant prostate cancer

**DOI:** 10.1002/cam4.4172

**Published:** 2021-08-12

**Authors:** Cedric Pobel, Edouard Auclin, Diego Teyssonneau, Brigitte Laguerre, Mathilde Cancel, Elouen Boughalem, Johanna Noel, Pierre Emmanuel Brachet, Denis Maillet, Philippe Barthelemy, Carole Helissey, Constance Thibault, Stéphane Oudard

**Affiliations:** ^1^ Oncology Department Hôpital Européen Georges Pompidou AP‐HP University of Paris Paris France; ^2^ Oncology Department Bergonié Institut Bordeaux France; ^3^ Oncology Department Eugène Marquis Center Rennes France; ^4^ Oncology Department University hospital of Tours France; ^5^ Oncology Department Institut de Cancérologie de l’Ouest Angers France; ^6^ Oncology Department Centre François Baclesse Caen France; ^7^ Oncology Department University hospital of Lyon France; ^8^ Medical Oncology University Hospital Strasbourg / Institut de Cancérologie Strasbourg Europe Strasbourg France; ^9^ Clinical Research Unit Military Hospital Begin Saint Mandé France

**Keywords:** prostate cancer, chemotherapy, metastasis, cancer management, urological oncology

## Abstract

**Introduction:**

Cabazitaxel multiple rechallenges may be a treatment option in heavily pretreated patients with metastatic castration‐resistant prostate cancer (mCRPC) who had a good initial response to cabazitaxel and who are still fit to receive it. Our objective was to assess the efficacy and toxicity of multiple rechallenges.

**Patients and methods:**

We retrospectively identified 22 mCRPC patients previously treated with docetaxel and/or androgen receptor‐targeted agents who received multiple cabazitaxel rechallenges in 9 French centers. Cabazitaxel was initiated at a dose of 25 mg/m^2^ q3week. A reduced dose (20 mg/m^2^ q3w) or an alternative schedule (mainly 16 mg/m^2^ q2w) was increasingly used for subsequent rechallenges. Progression‐free survival, prostate‐specific antigen (PSA) response, best clinical response, and grade ≥3 toxicities were collected. Overall survival was calculated from various time points.

**Results:**

Twenty‐two patients with an initial response to cabazitaxel were rechallenged at least twice. The median number of cabazitaxel cycles was 7 at first cabazitaxel treatment, 6 at first rechallenge, and 5 at subsequent rechallenges. Median progression‐free survival at first rechallenge was 9.6 months and 5.6 months at second rechallenge. Median overall survival was 50.9 months from the first cabazitaxel dose, 114.9 months from first life‐extending therapy initiation in mCRPC, and 105 months from mCRPC diagnosis. There was no cumulative grade ≥3 neuropathy or nail disorder and one case of febrile neutropenia.

**Conclusion:**

Cabazitaxel multiple rechallenges may be a treatment option without cumulative toxicity in heavily pretreated patients having a good response to first cabazitaxel use and still fit to receive it.

**Novelty & Impact Statements:**

Patients with metastatic castration‐resistant prostate cancer can be treated with Cabazitaxel after docetaxel and androgen receptor‐targeted agent. This chemotherapy can be used multiple times with efficacy and manageable toxicity.

## Graphical abstract

We assessed the efficacy and toxicity of multiple rechallenges with cabazitaxel for 22 patients with metastatic castration‐resistant prostate cancer (mCRPC). Median overall survival was 50.9 months from the first cabazitaxel dose, 114.9 months from first life‐extending therapy initiation in mCRPC, and 105 months from mCRPC diagnosis. Cabazitaxel rechallenge may be an effective option in heavily pretreated mCRPC patients with a good initial response to cabazitaxel and still fit to receive it, without cumulative grade ≥3 toxicity.

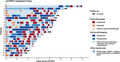

## 1 INTRODUCTION

Since 2004, several agents have shown an overall survival (OS) benefit in metastatic castration‐resistant prostate cancer (mCRPC) patients, including in chronological order docetaxel, sipuleucel‐T cabazitaxel, abiraterone acetate, enzalutamide, radium‐223, and olaparib.^1^, ^2^ Cabazitaxel use is restricted to patients previously treated with docetaxel,^3^, ^4^ olaparib use is restricted to patients with BRCA1/2 mutations previously treated with an androgen receptor‐targeted agent (ARTA)^2^ and there is now level 1 evidence that patients who have progressed with a first ARTA poorly respond to another ARTA.^2^, ^5^, ^6^ Treatment options in mCRPC are thus limited.

Cabazitaxel is a next‐generation taxane that retains its activity in mCRPC patients progressing on docetaxel^3^ or ARTA.^6^, ^7^, ^8^ In the prospective CARD trial, cabazitaxel significantly improved radiographic progression free‐survival (PFS) and OS versus abiraterone or enzalutamide in mCRPC patients who had received docetaxel and progressed within 12 months with the alternative ARTA.^6^ Two large retrospective registries conducted by our institution in daily life suggested that a treatment sequence including docetaxel, one ARTA, and cabazitaxel provided an optimal OS.^9^, ^10^ The main toxicity of cabazitaxel is the risk of neutropenia which can be effectively prevented by G‐CSF prophylaxis from the first cycle.^6^ Compared to docetaxel, cabazitaxel also shows a reduced incidence of alopecia, peripheral neuropathy, peripheral edema, and nail disorders with less cumulative toxicity.^4^, ^11^ Rechallenge with cabazitaxel could thus be an interesting option for mCRPC patients having exhausted available life‐extending therapies.

Here, we report the efficacy and toxicity of multiple cabazitaxel rechallenges in heavily pretreated mCRPC patients in daily practice.

## 2 MATERIALS AND METHODS

### 2.1 Setting and design

We retrospectively reviewed clinical data of 710 consecutive mCRPC patients treated with cabazitaxel in nine French centers from February 2012 to July 2020. Of them, 22 were rechallenged at least twice with cabazitaxel.

Disease history was collected for each patient. The activity of each cabazitaxel treatment line was measured by prostate‐specific antigen (PSA) response ≥50% from baseline, best clinical benefit (as per investigator judgment based on European Cooperative Oncology Group performance status [ECOG‐PS], pain, and analgesic consumption), radiological and/or clinical PFS. OS was calculated from mCRPC diagnosis, from initiation of first life‐extending therapy, first cabazitaxel treatment, and each cabazitaxel rechallenge. Progressive disease was defined by two on the three following criteria of the Prostate Cancer Clinical Trials Working Group (PCWG2): Radiological progression according to RECIST 1.1, PSA rising ≥ 25% or clinical progression. Grade ≥3 toxicities according to the Common Terminology Criteria for Adverse Events (CTCAE) version 5 were collected. Follow‐up for all deceased patients was complete until the time of their death.

### 2.2 Ethics

This study received Ethics Committee approval. Patients still alive gave their written consent and confidentiality approval was obtained from the Commission Nationale de l’Informatique et des Libertés (CNIL).

### 2.3 Data analyses

Analyses were descriptive and conducted using the R^®^ software version 3.6.1. OS and PFS were estimated by the Kaplan–Meier method.

## 3 RESULTS

### 3.1 Patient characteristics

**FIGURE 1:**
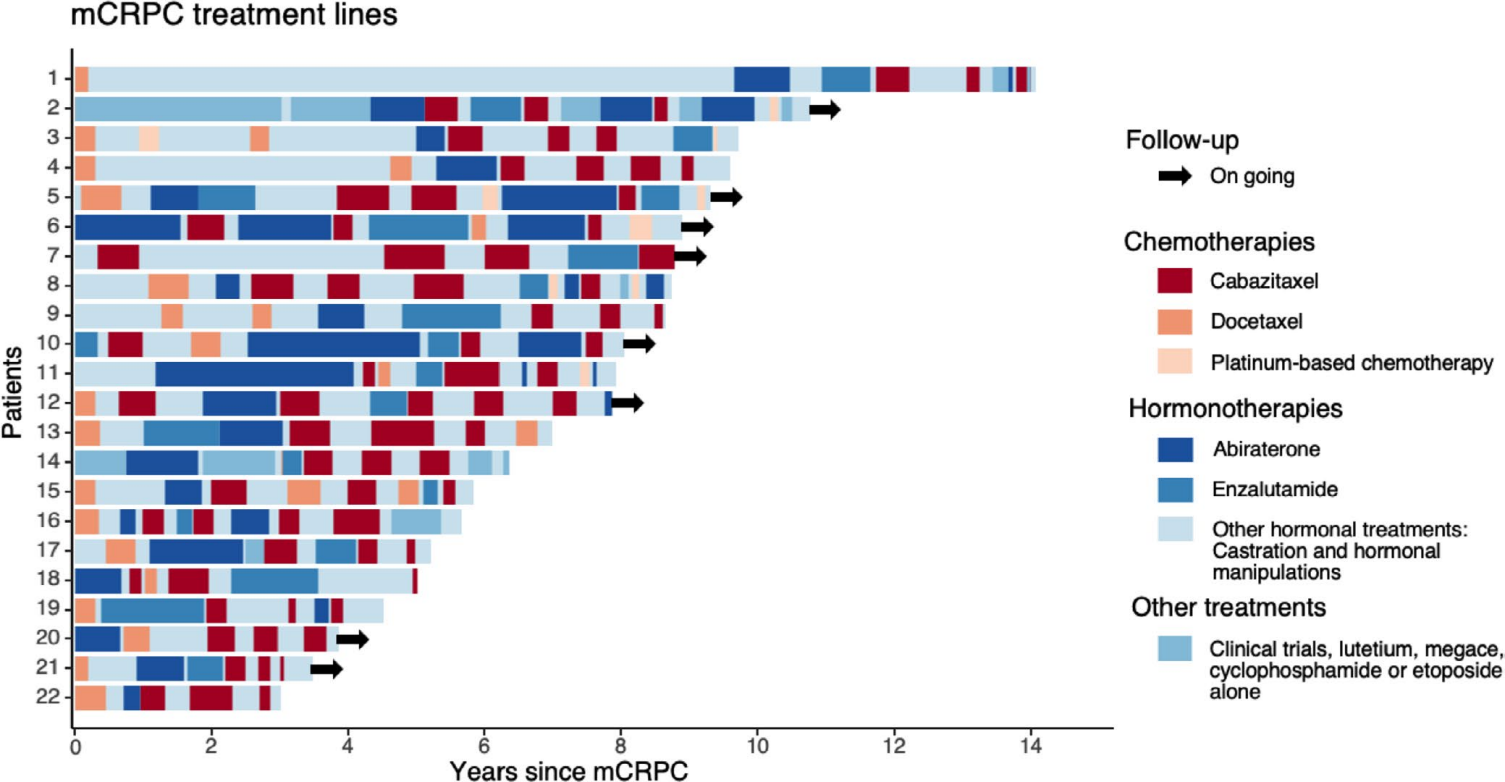
Swimmer plot summarizing treatment strategies from the diagnosis of mCRPC for each patient rechallenged with cabazitaxel. mCRPC: metastatic castration‐resistant prostate cancer

Median follow‐up from mCRPC diagnosis for the 22 mCRPC patients rechallenged with cabazitaxel was 94.7 months. Almost all patients had received docetaxel and at least one ARTA before cabazitaxel initiation (Figure 1). Treatment sequence did not include sipuleucel T, radium‐223 or olaparib because these therapies are not reimbursed in France. Cabazitaxel was initiated in third‐line setting or beyond in 17 patients (77.3%). Clinical characteristics are provided in Table 1.

**TABLE 1:** Clinical characteristics of 22 mCRPC patients rechallenged with cabazitaxel

Characteristics	No. (%)
Total number of patients	22
Median age at first treatment initiation (range), years	60 (52–78)
Gleason score 8–10 at diagnosis, n (%)	12 (57.1)
Median follow‐up from mCRPC diagnosis, months	94.7
Characteristics at cabazitaxel initiation	
Metastatic sites (Halabi classification), n (%)	
Lymph node only	0
Bone (+/‐ lymph nodes)	19 (86.4)
Visceral (+/‐ bone, +/‐ lymph nodes)	3 (13.6)
ECOG PS 0–1, n (%)	19 (86.4)
Pain, n (%)	13 (59.1)
Consumption of narcotic analgesics, n (%)	6 (27.3)
Biology	
Median PSA, ng/ml	85.7
Median hemoglobin, g/dl	12.0
Median neutrophil to lymphocyte ratio	3.3
LDH >ULN, n (%)	2 (22.2)
ALP >ULN, n (%)	5 (50)

Abbreviations: ALP, Alkaline phosphatase; ECOG‐PS, European Cooperative Oncology Group performance status; LDH, Lactate dehydrogenase; mCRPC, metastatic castration‐resistant prostate cancer; PSA, prostate‐specific antigen.

### 3.2 Cabazitaxel treatment

Patients rechallenged with cabazitaxel were good responders to first cabazitaxel use, in terms of PSA response, clinical benefit, and PFS (Table 2). Overall patients received a median number of 19.5 cabazitaxel cycles (7 at first use, 6 at first rechallenge, and 5 at subsequent rechallenges). In most cases, cabazitaxel was initiated at a dose of 25 mg/m^2^ every 3 weeks but a reduced dose (20 mg/m^2^ every 3 weeks) or an alternative schedule (mainly 16 mg/m^2^ every 2 weeks) was increasingly used for subsequent rechallenges. Median cabazitaxel‐free interval decreased with subsequent rechallenges. Most patients received prophylactic G‐CSF.

**TABLE 2:** Efficacy and toxicity of each cabazitaxel treatment in 22 mCRPC patients rechallenged with cabazitaxel

Patient (N)	First use (n = 22)	Second use (n = 22)	Third use (n = 22)	Fourth use (n = 5)	Fifth use (n = 1)
**Cabazitaxel initial dose, N (%)**					
25 mg/m^2^ q3w	16 (72.7)	8 (36.4)	2 (9.1)	1 (20)	0
20 mg/m^2^ q3w	3 (13.6)	5 (22.7)	10 (45.5)	1 (20)	0
16 mg/m^2^ q2w	3 (13.6)	9 (40.9)	9 (40.9)	3 (60)	1
10 mg/m^2^ weekly	0	0	1 (4.55)	0	0
**Median number of cycles, N (range)**	7 (4–12)	6 (3–16)	5 (1–12)	5 (3–12)	5
**Prophylactic G‐CSF, N (%)**	14 (63.6)	18 (81.8)	17 (77.3)	4 (80)	1
**Best clinical benefit, N (%)**					
Improved	11 (50)	11 (50)	10 (45.5)	1 (20)	1
Stable	10 (45.5)	11 (50)	7 (31.8)	4 (80)	0
Worse	1 (4.5)	0	5 (22.7)	0	0
Disease control rate	21 (95.5)	22 (100)	17 (77.3)	5 (100)	1
**PSA response (%)**					
≥50%	19 (86.4)	14 (63.6)	12 (60)	4 (80)	0
**PFS from each line start**					
Median (months)	11.8	9.6	5.6	10.2	9.1
[95% CI]	[9–14.2]	[8.6–12.2]	[4.2–9]	[7‐NR]	—
**Overall survival from each line start**					
Median (months)	50.9	43.5	24.8	19.2	—
[95% CI]	[44.5‐NR]	[26.9‐NR]	[13.7‐NR]	[8.3‐NR]	
**Reason for discontinuation, N (%)**					
Predefined number of courses	21 (95.5)	18 (81.8)	10 (45)	2 (40)	1
Progression	0	1 (4.5)	10 (45)	1 (20)	0
Impaired ECOG PS	1 (4.5)	2 (9.1)	2 (9.1)	1 (20)	0
Toxicity	0	1 (4.5)	0	1 (20)	0
**Free interval between cabazitaxel‐line use**	9.5	6.9	7.2	8.6	—
Median in month (range)	(2.3–56.0)	(1.6–41.5)	(3.7–20.6)	—	—
Toxicity ≥grade 3 (N)					
Fatigue	2	0	0	0	0
Diarrhea	3	0	1	0	0
Hematuria	0	1	2	0	0
Febrile neutropenia	0	0	1	0	0
Cholestasis	0	1	0	0	0

Abbreviations: ECOG‐PS, European Cooperative Oncology Group performance status; G‐CSF, granulocyte‐colony stimulating factor; NR, not reached; PFS, progression‐free survival; PSA, prostate‐specific antigen.

A clinical benefit measured at its best was observed in 50% and 45% of patients at first and second rechallenges. A PSA decrease of at least 60% was observed at first, second, and third cabazitaxel rechallenges. Median PFS was 11.8 months at first cabazitaxel use, 9.6 months at first rechallenge, and 5.6 months at second rechallenge. Median OS reached 105 months from mCRPC diagnosis, 114.9 months from first life‐extending therapy, 50.9 months from first cabazitaxel use, 43.5 months from the first rechallenge, 24.8 months from the second rechallenge, and 19.2 months from the third rechallenge. The median number of cycles received was 7 (4 to 12), 6 (3 to 16), 5 (1 to 12), 5 (3 to 12), and 5 at first, second, third, fourth, and fifth use of cabazitaxel, respectively. The reason for treatment discontinuation was the completion of the predefined number of cabazitaxel cycles for most of the patients: 95.5% of them at first use, 81.8% of them at second use, 45% of them at third use, and 50% of them at fourth use. Progressive disease caused discontinuation in 4.5% of cases at second use, 45% of cases at third use, and 25% of cases at fourth use. Toxicity ≥grade 3 was found to cause treatment discontinuation for only one patient at second and fourth use.

The toxicity of cabazitaxel was manageable with only one case of febrile neutropenia during the second rechallenge. Six patients experienced grade ≥3 adverse events during the first or second rechallenge (hematuria, n = 3; cholestasis n = 1, diarrhea, n = 1, febrile neutropenia, n = 1). No grade ≥3 neuropathy or alopecia or nail disorders was reported.

## 4 DISCUSSION

This is, to our knowledge, the first report of multiple cabazitaxel rechallenges in mCRPC patients treated in daily practice. Key findings of our research are those good responders to the first use of cabazitaxel, even when heavily pretreated, may be rechallenged several times with cabazitaxel with a good response to therapy and without evidence of cumulative toxicity. Indeed, in those 22 mCRPC patients analyzed, we showed a median PFS of 9.6 months and 5.6 months and a disease control rate of 100% and 77.3% at first and second cabazitaxel rechallenge, respectively. OS calculated from first life‐extending therapy and mCRPC diagnosis reached 114.9 and 105 months, respectively. The most common grade ≥3 adverse events was the occurrence of hematuria (three patients) and one patient reported febrile neutropenia. There was no cumulative grade ≥3 peripheral neuropathy or nail disorder.

Our results further support those previously reported by Thibault et al in 69 mCRPC patients well responding to cabazitaxel and rechallenged once with the same drug.^12^ Median PFS with cabazitaxel rechallenge was 7.8 months and a best clinical benefit which was improved in 34.3% and stable in 47.8% of cases. Median OS calculated from the first life‐extending therapy and from mCRPC diagnosis reached 59.9 months and 78.3 months, respectively. No cumulative toxicity was reported.

We previously reported that docetaxel could be rechallenged in mCRPC patients having a good initial response.^13^ However, docetaxel rechallenge is associated with a cumulative incidence of peripheral neuropathy, nail disorders, and asthenia/fatigue which is bothersome for the patients. Moreover, there is increasing evidence that docetaxel may lose activity in patients previously treated with ARTA.^7^, ^9^ This may be due to a restauration of androgens in microenvironment after ARTA was found because of all the mechanisms of resistance restoring androgen resulting in tumor progression. This androgen level in microenvironment could explain docetaxel lost of activity due to difficulties for tumoral penetration.^14^ Cabazitaxel shows a greater intra‐tumoral penetration than docetaxel^15^ and has been shown in preclinical models and prospective clinical studies to retain its activity in patients who have progressed with ARTA.^6^, ^8^

Retrospective design with a small sample of patients is the main limitation of our study. It is also important to note that patients analyzed were highly selected since they were good responders to cabazitaxel initial treatment and were fit enough to be rechallenged with it. Nonetheless, our results are promising and should be confirmed by prospective clinical studies.

## 5 CONCLUSION

In conclusion, our results suggest that, in heavily pretreated mCRPC patients, cabazitaxel can be rechallenged without cumulative toxicity in mCRPC patients with good initial response to cabazitaxel and still fit to receive it. In this selected population, cabazitaxel multiple rechallenges may also extend OS with no cumulative toxicity. Cabazitaxel rechallenge should thus be considered as a therapeutic option for such patients.

## ETHICAL APPROVAL STATEMENT

This study received Ethics Committee approval. Patients still alive gave their written consent and confidentiality approval was obtained from the Commission Nationale de l’Informatique et des Libertés (CNIL).

## AUTHOR CONTRIBUTIONS

Conception and design: Constance Thibault & Stéphane Oudard. Acquisition of data: Cedric Pobel, Diego Teyssonneau, Brigitte Laguerre, Mathilde Cancel, Elouen Boughalem, Johanna Noel, Pierre Emmanuel Brachet, Denis Maillet, Philippe Barthelemy, Carole Helissey. Statistical analysis and interpretation of data: Cedric Pobel & Edouard Auclin. Drafting of the manuscript: Cedric Pobel & Edouard Auclin. Critical revision of the manuscript for important intellectual content: Elouen Boughalem, Johanna Noel, Pierre Emmanuel Brachet, Denis Maillet, Philippe Barthelemy, Carole Helissey & Constance Thibault. Supervision: Constance Thibault & Stéphane Oudard.

## CONFLICT OF INTEREST

The authors have no conflict of interest to declare.

## Data Availability

The research data are available upon reasonable request to the corresponding author (cedric.pobel@gmail.com).
